# Supplementary material for: Expression of Periostin Alternative Splicing Variants in Normal Tissue and Breast Cancer
Source: Biomolecules. 2024 Aug 31;14(9):1093. doi: 10.3390/biom14091093 (PMC11430663; doi:10.3390/biom14091093)
Supplement: Supplementary file 1 [file biomolecules-14-01093-s001.zip › biomolecules-3116556 supplent information.pdf]

Supplementary information

Expression of Periostin Alternative Splicing Variants in Normal Tissue and Breast Cancer

Yuko Kanemoto [1], Fumihiro Sanada [2], Kana Shibata [3], Yasuo Tsunetoshi [2], Naruto Katsuragi [2], Nobutaka Koibuchi [2], Tetsuhiro Yoshinami [1], Koichi Yamamoto [4], Ryuichi Morishita [2], Yoshiaki Taniyama [3] and Kenzo Shimazu [1,*]

[1] Department of Breast and Endocrine Surgery, Osaka University Graduate School of Medicine,
Suita 565-0871, Japan; y.kanemoto@onsurg.med.osaka-u.ac.jp (Y.K.); yosinami-te@onsurg.med.osaka-u.ac.jp (T.Y.)

[2] Department of Clinical Gene Therapy, Osaka University Graduate School of Medicine,
Suita 565-0871, Japan; sanada@cgt.med.osaka-u.ac.jp (F.S.); tsunetoshi@geriat.med.osaka-u.ac.jp (Y.T.); katsuragi@cgt.med.osaka-u.ac.jp (N.K.); koibuchi@cgt.med.osaka-u.ac.jp (N.K.); morishit@cgt.med.osaka-u.ac.jp (R.M.)

[3] Department of Advanced Molecular Therapy, Osaka University Graduate School of Medicine,
Suita 565-0871, Japan; shibata@cgt.med.osaka-u.ac.jp (K.S.); taniyama@cgt.med.osaka-u.ac.jp (Y.T.)

[4] Department of Geriatric and General Medicine, Osaka University Graduate School of Medicine,
Suita 565-0871, Japan; kyamamoto@geriat.med.osaka-u.ac.jp

* Correspondence: kshimazu@onsurg.med.osaka-u.ac.jp; Tel.: +81-6-6879-3772; Fax: +81-6-6879-3779

Running title: Periostin alternative splicing variants

Supplementary Figure legends

Supplement figure 1. Periostin ASVs in mouse and human.

In mouse and human, Pn undergoes alternative splicing in its c-terminal region, which lacks known functional domains. Four ASVs of mouse Pn (mPn) and eight for human Pn (hPn) have been reported. The image shows a simplified diagram of Pn, which comprises cysteine-rich domain (EMI), four fasciclin 1 domains (FAS1), and c-terminal domain. Number of exon of Pn c-terminal from 15 to 23 are shown. Gene accession numbers were also shown.

Supplement figure 2. Physiological distribution of mPn-ASVs protein in adult mice.

A. Confirmation of total mPn protein expression in adult mouse organs. Total mPn protein expression was measured separately from cerebrum and skeletal muscle by immunoblotting with Pn exon 12 Ab. Supernatant from human Pn-1-Halotag overexpressed HEK293T cells (10 and 20μg) and mouse 4T1 breast cancer cells (20 and 40μg) were simultaneously blotted as a positive control. Proteins from Pn KO mice were used as negative control. N=3 for each organ. B. IP-western blotting for mPn-ASVs. 75μg tissue proteins from lung and colon were immunoprecipitated with exon 14 (lane 2), 17 (lane 3), 21 (lane 4) and control IgG (lane 5), Immune complexes were collected on protein G-Agarose beads under agitation. Proteins were solubilized in Laemmli buffer, separated by SDS-PAGE, transferred to PVDF membranes and detected with Pn exon 12 Ab. 10μg un-precipitated sample was also loaded to see the molecular level of physiologically expressed mPn-ASVs in lane 1. Lane 6 shows beads only. Coomassie brilliant blue (CBB) staining was performed according to the manufacture's instruction.

Supplement figure 3. ISH against total hPn and hPn-ASVs exon 21 in ovarian cancer and laryngeal cancer.

A and C. ISH with probe for total hPn. Peri-tumoral stroma cells showed strong positive signals both in ovarian cancer and laryngeal cancer samples. B and D. ISH with probe for hPn-ASVs with exon 21. Peri-tumoral stroma cells with spindle shaped nuclei similarly showed strong positive signals both in ovarian cancer and laryngeal cancer samples.

Supplement figure 4. mPn-ASVs mRNA expression in 4T1, 4T07 mouse TNBC cell lines.

Expression level of mPn-ASVs mRNA in 4T1 and 4T07 mouse TNBC cells were measured by RT-PCR with variant specific primers. Data shown as mean ± SE, n=6,

*P≤0.05 vs 4T1 cell.

Supplement figure 5. Pn-21Ab inhibited the growth of 4T07 syngeneic mouse model by suppressing several cytokines and chemokines.

A In total, $1x10^5$ cells from 4T07 cells were injected into the abdominal mammary glands of female Balb/c mice. Mice bearing 4T07 cell tumors were treated with Pn-21Ab (30 mg/kg) or vehicle, 2 times per week throughout the experiments starting at day 6 (n=5 each). When mice were sacrificed, serum were collected and mixed from 5 mice in each group. Several cytokines and chemokines were comprehensively examined using the Proteome Profiler Mouse XL Cytokine Array to investigate the mechanism of drug efficacy of Pn-21Ab on breast cancer. From the results obtained, the tumor-induced increase in serum cytokines/chemokines and the inhibitory effect of Pn-21Ab were calculated. w/o; without, w/; with
