# Supplementary material for: Expression of Periostin Alternative Splicing Variants in Normal Tissue and Breast Cancer
Source: Biomolecules. 2024 Aug 31;14(9):1093. doi: 10.3390/biom14091093 (PMC11430663; doi:10.3390/biom14091093)
Supplement: Supplementary file 1 [file biomolecules-14-01093-s001.zip › Supplement figure.pdf]

Figure

- Supplement figure 1-5

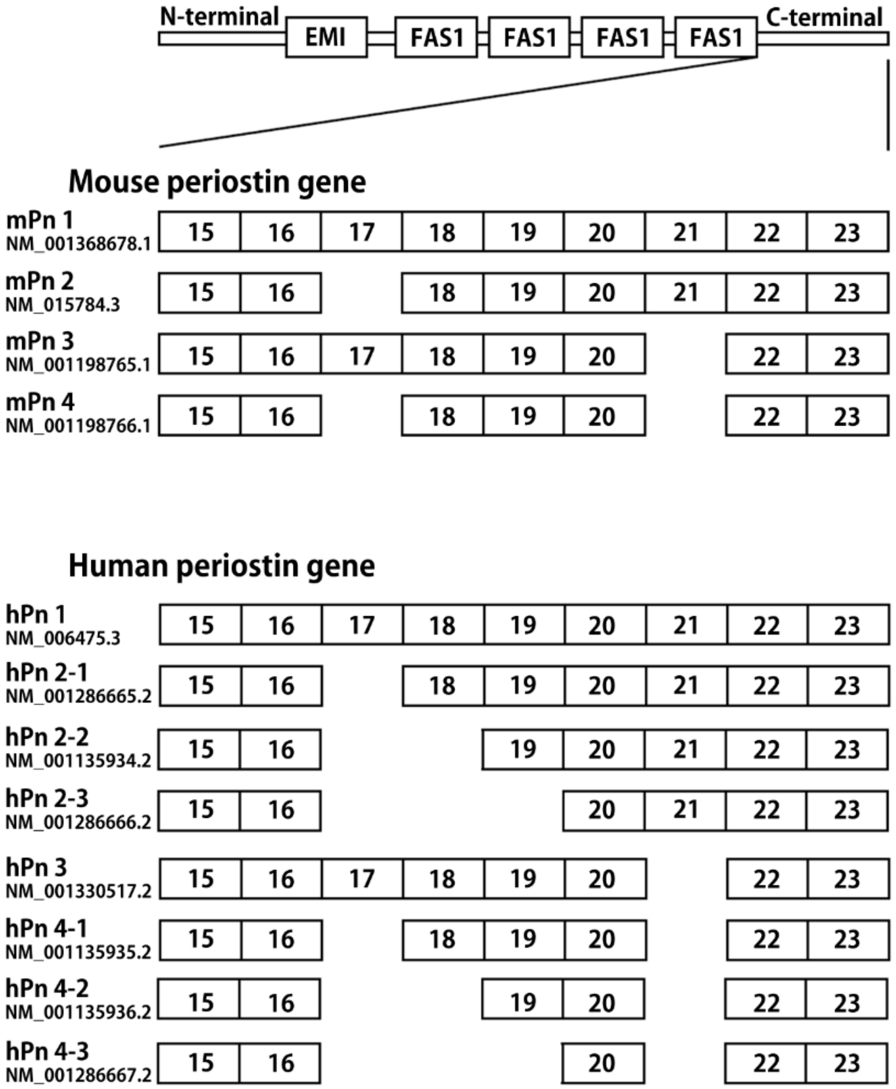

Supplement figure 1

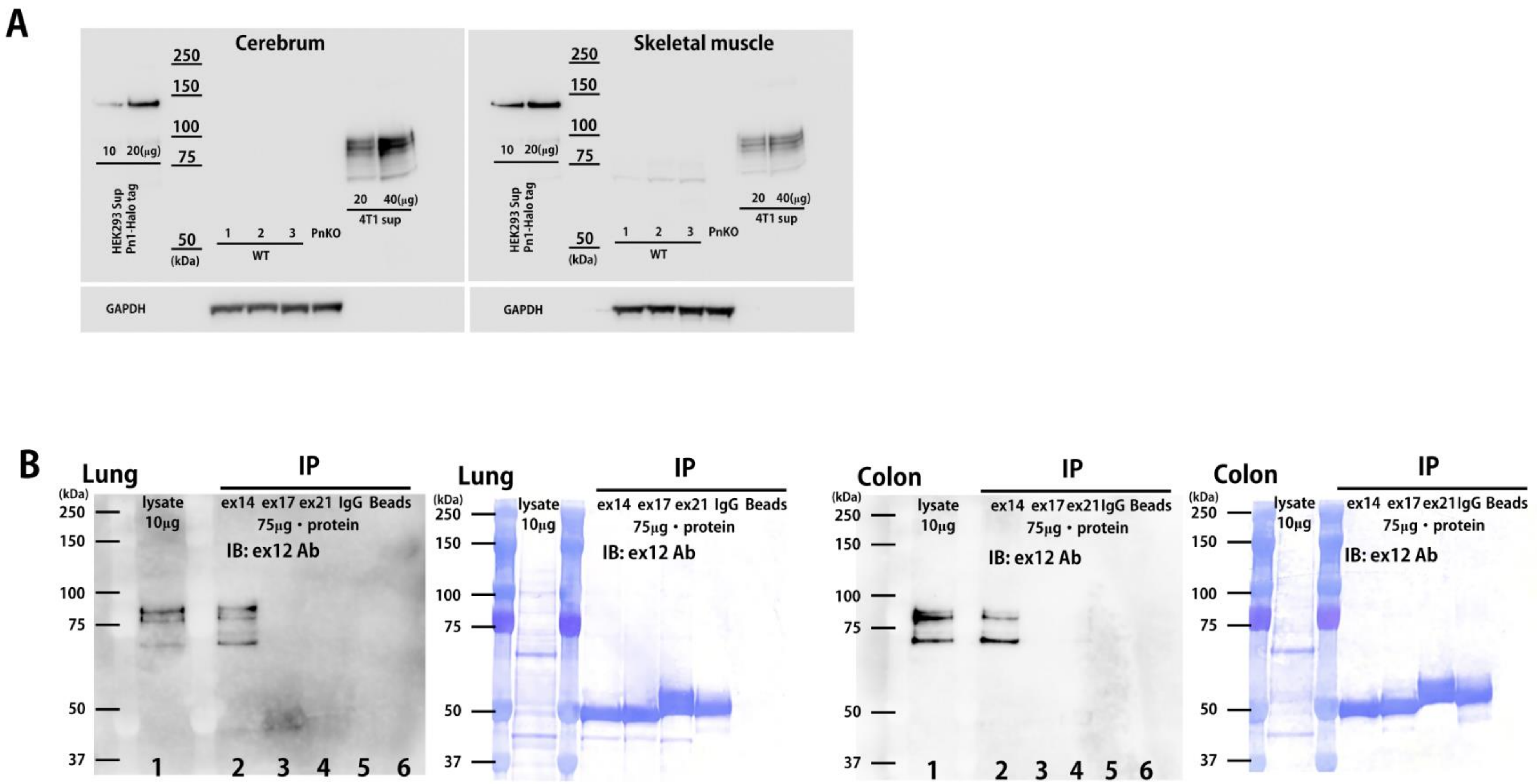

Supplement figure 2

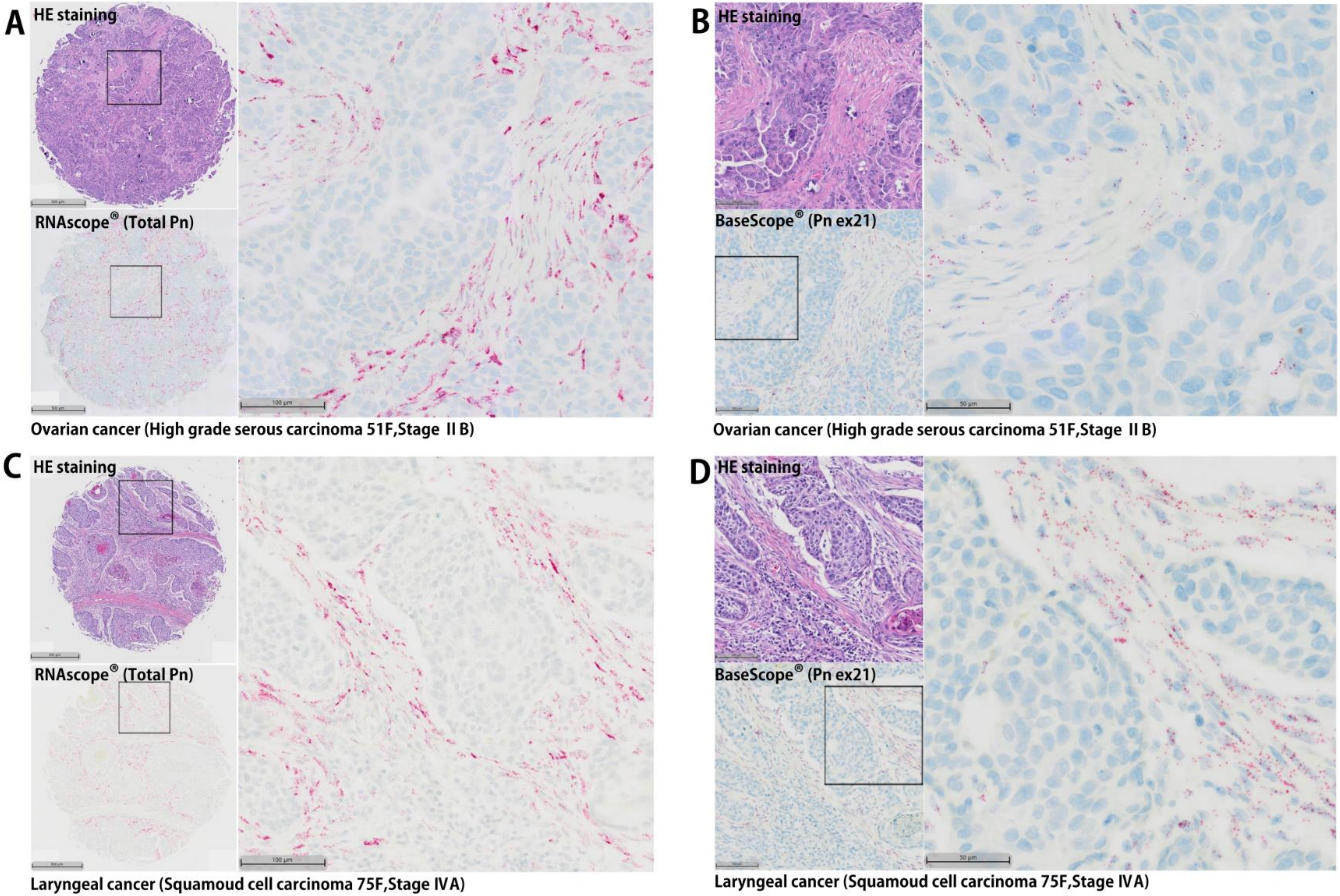

Supplement figure 3

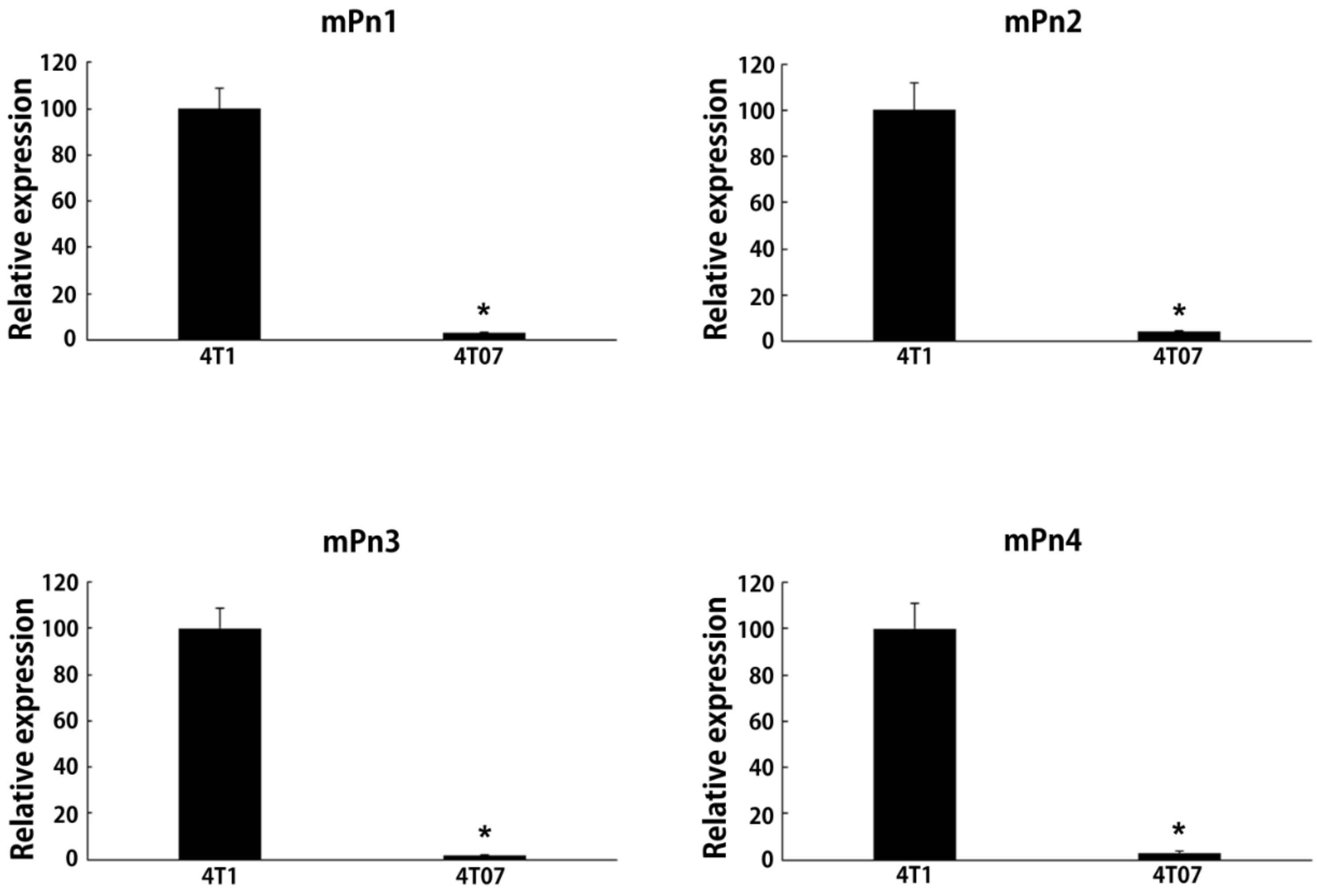

Supplement figure 4

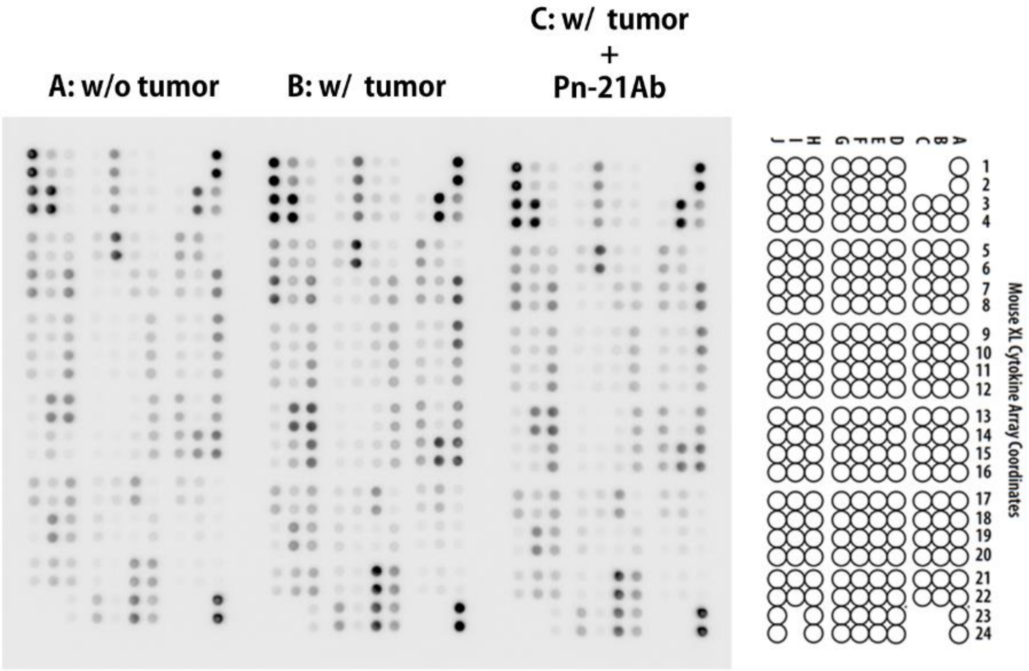

Coodinate	Analyte	A: w/o tumor	B: w/ tumor	C: Pn-21Ab	Effects of Tumors: B/A	Suppression by Pn-21Ab (%): (B-C)/(B-A)*100
H15, 16	MMP-9	13.5	67.8	54.9	5.01	23.8
E9, 10	G-CSF	12.0	26.6	17.3	2.22	63.6
E21, 22	IGFBP-1	44.6	94.7	80.4	2.12	28.6
D7, 8	CXCL13/BLC/BCA-1	20.8	42.8	28.0	2.06	67.4
B7, 8	CCL12/MCP-5	17.3	31.6	21.4	1.83	71.5
B17, 18	CCL22/MDC	19.2	34.5	23.6	1.79	71.7
H21, 22	Osteoprotegerin/TNFRSF11B	22.6	37.8	28.1	1.67	64.0
E3, 4	FGF-21	14.6	23.1	18.9	1.58	49.8
J7, J8	Serpin E1/PAI-1	46.4	73.4	56.1	1.58	63.9
I1, I2	PDGF-BB	31.2	49.2	27.5	1.58	120.7
B15, 16	CCL21/6Ckine	51.3	80.9	62.4	1.58	62.6
B3, B4	CCL6/C10	74.3	116.8	94.5	1.57	52.5
C7, C8	Chitinase 3-like 1	27.9	41.4	33.7	1.49	57.2
F3, F4	IGFBP-5	43.3	63.6	45.2	1.47	90.9
F1, F2	IGFBP-3	49.1	71.5	54.3	1.46	77.0
F21, 22	IL-6	9.7	14.1	11.4	1.45	61.4
F11, 12	IL-1ra/IL-1F3	11.6	16.3	13.8	1.41	52.2
C11, C12	Complement Component C5/C5a	17.7	24.9	19.9	1.41	70.2
E1, 2	FGF acidic	11.8	16.6	12.8	1.40	80.1
A17, 18	CCL2/JE/MCP-1	10.9	15.4	12.8	1.40	58.8
I5, I6	Pentraxin 3/ TSG-14	20.3	28.4	28.1	1.40	3.1
G23, G24	LDL R	32.0	44.7	34.9	1.40	77.1
B13, 14	CCL20/MIP-3 alpha	12.2	17.0	12.8	1.39	86.4
B21, B22	CD40/TNFRSF5	11.3	15.5	13.2	1.38	53.6
D22, D23	Fetuin A/AHSG	31.5	43.3	35.2	1.37	69.3
E13, E14	GM-CSF	10.9	14.9	13.5	1.37	34.4
F15, 16	IL-3	10.8	14.8	11.7	1.37	78.7
E11, E12	GDF-15	11.8	16.1	14.1	1.37	48.2
H13, H14	MMP-3	58.0	78.9	70.4	1.36	40.7
D9, 10	CXCL16	28.5	38.7	33.2	1.36	53.9
J5, J6	P-Selectin/CD62P	35.8	48.5	44.2	1.35	33.4
I11, I12	Proliferin	13.9	18.6	16.5	1.34	44.7
B9, B10	CCL17/TARC	14.2	18.9	16.0	1.34	61.5
D3, D4	CXCL10/IP-10	13.5	17.9	14.5	1.33	77.8
G3, G4	IL-11	12.8	16.9	14.5	1.33	59.3
A7, A8	Angiopoietin-1	60.5	80.2	64.7	1.33	78.7
H3, H4	LIF	13.6	18.0	16.0	1.32	45.7
D1, D2	CXCL9/MIG	11.0	14.5	12.0	1.32	71.7
G1, G2	IL-10	11.2	14.7	12.6	1.31	60.6
G7, G8	IL-13	11.9	15.5	13.4	1.31	57.5
J3, J4	E-Selectin/CD62E	74.2	97.1	102.0	1.31	-21.1
F9, F10	IL-1 beta/IL-1F2	10.6	13.8	12.1	1.31	52.3
C3, C4	CD160	12.2	15.8	13.6	1.30	61.1
D21, 22	Endostatin	40.2	52.3	46.1	1.30	51.0
D5, D6	CXCL11/I-TAC	11.4	14.8	13.0	1.30	53.6

Supplement figure 5
