# Supplementary material for: Expression of Periostin Alternative Splicing Variants in Normal Tissue and Breast Cancer
Source: Biomolecules. 2024 Aug 31;14(9):1093. doi: 10.3390/biom14091093 (PMC11430663; doi:10.3390/biom14091093)

WB original image

Figure1

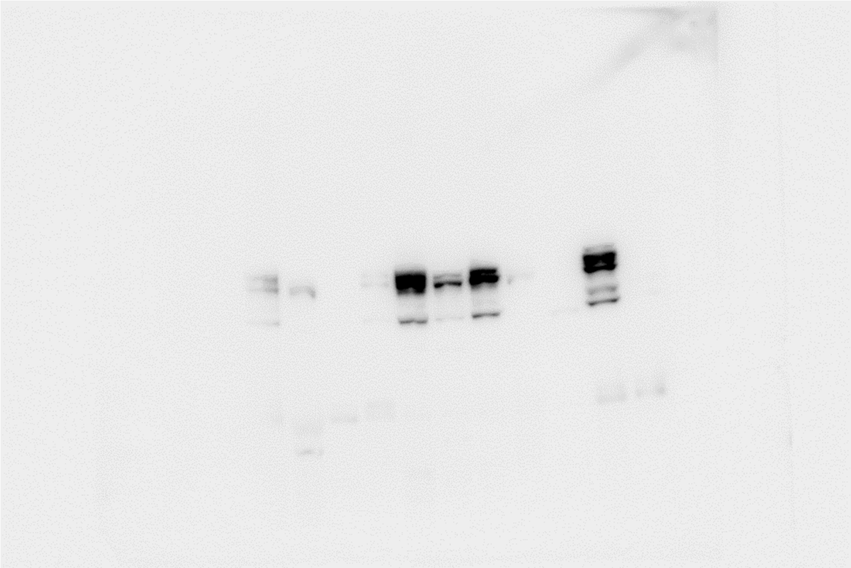

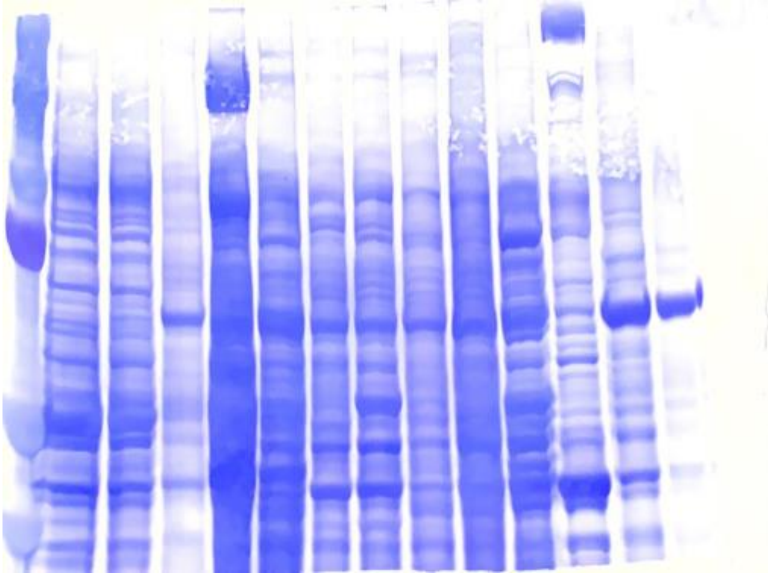

Figure2A

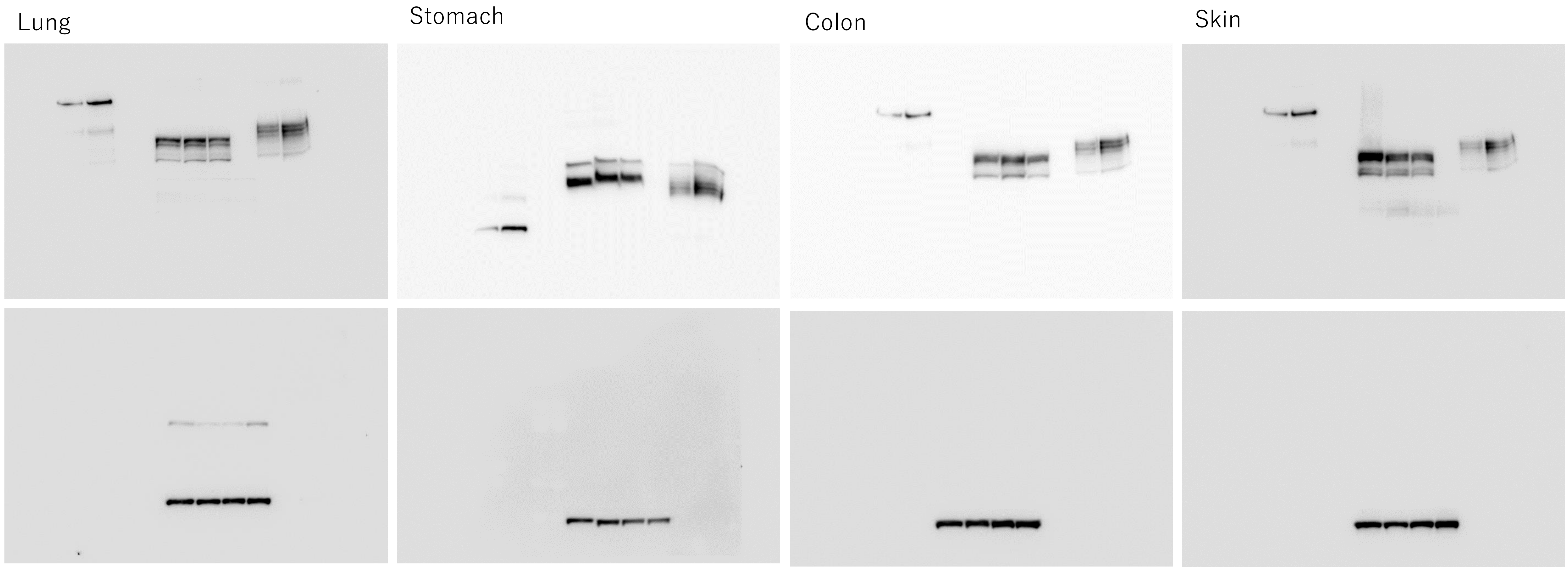

Figure2B, 2C

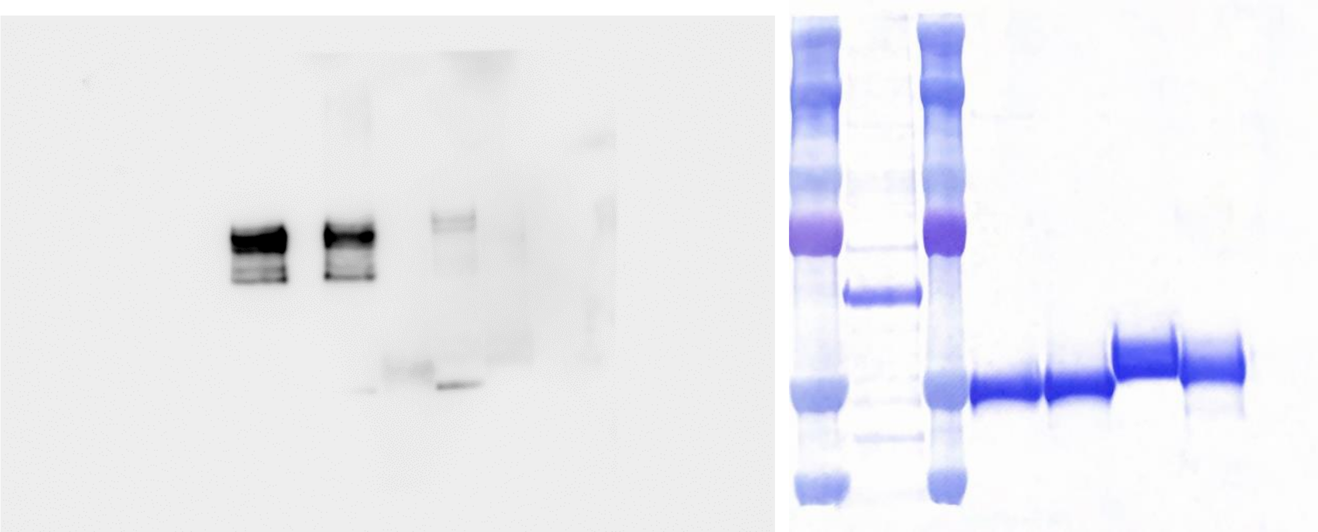

Figure2B

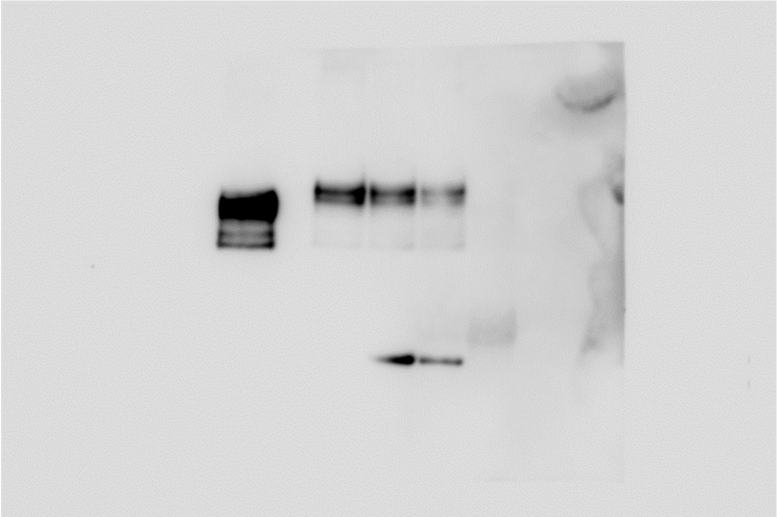

Figure2C

Figure4

Normal human Stomach

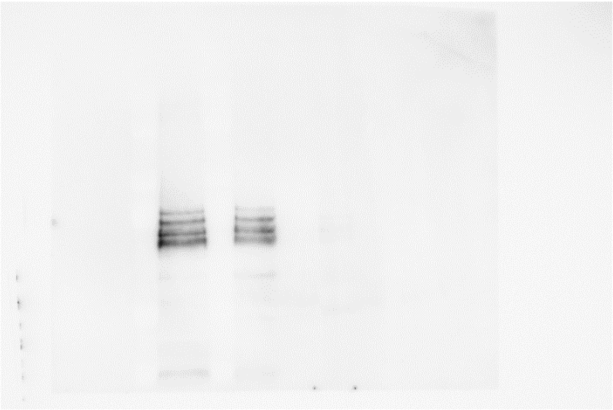

Normal human colon

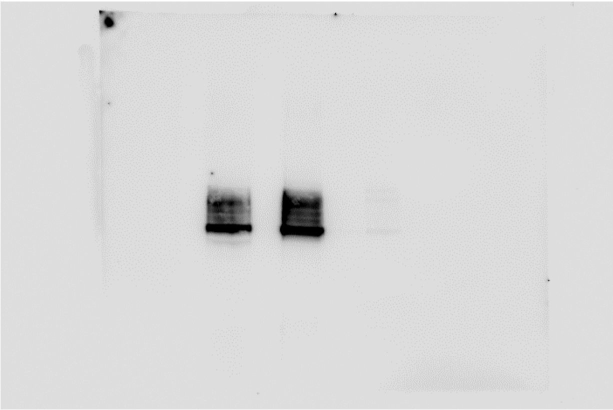

Normal human lung

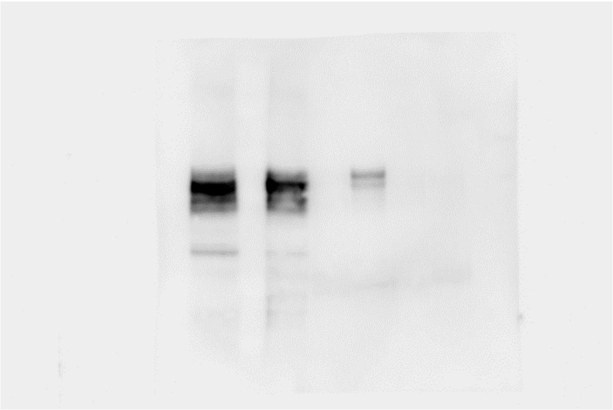

Normal human breast

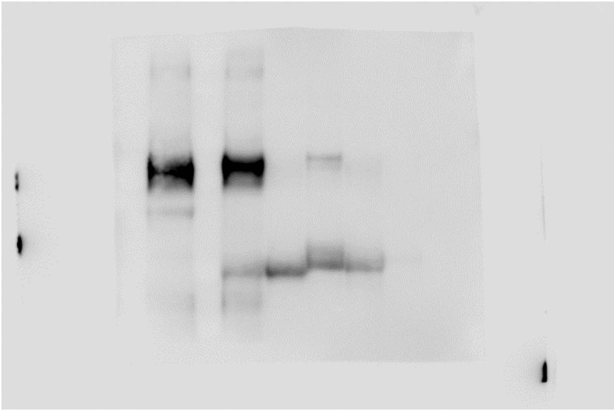

Figure5D

Pn-12Ab No.2.6.12

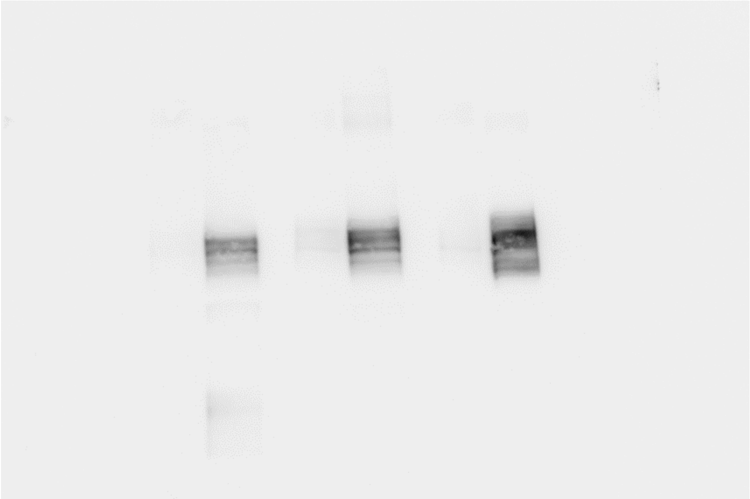

Pn-12Ab No.4.9.11

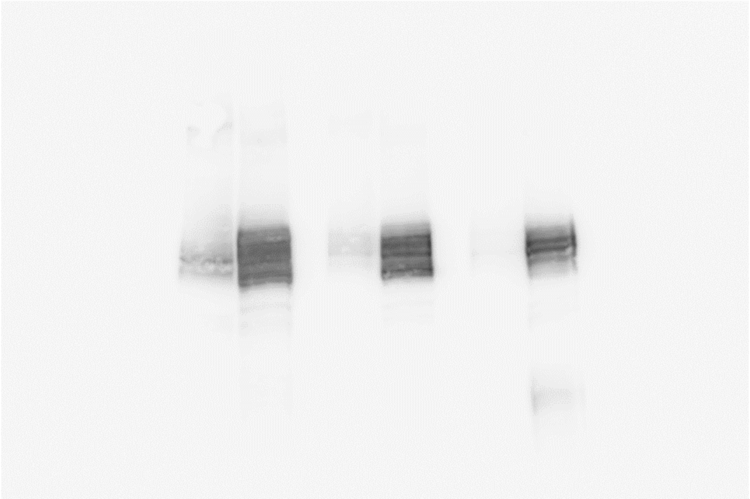

Pn-21Ab No.2.6.12

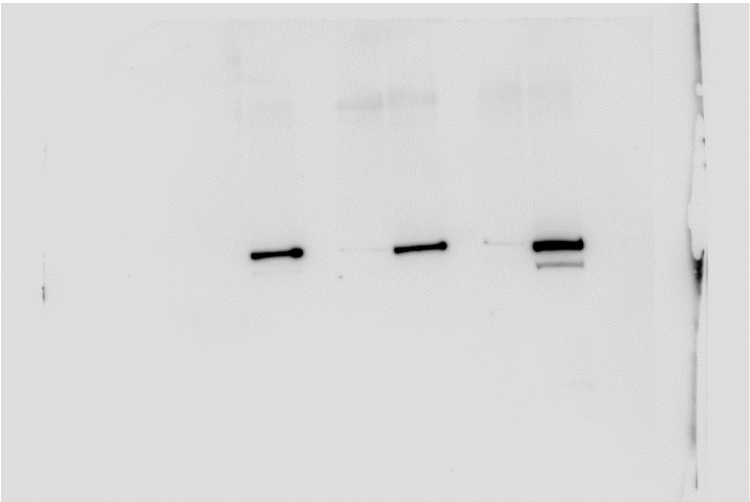

Pn-21Ab No.4.9.11

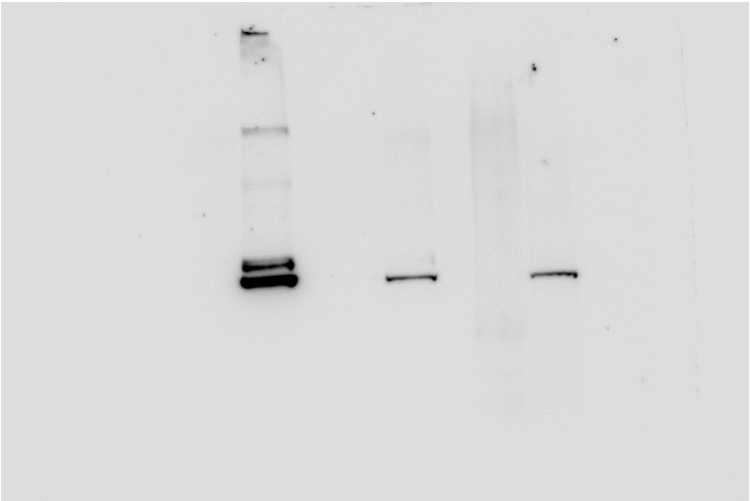

Supplement Figure2A

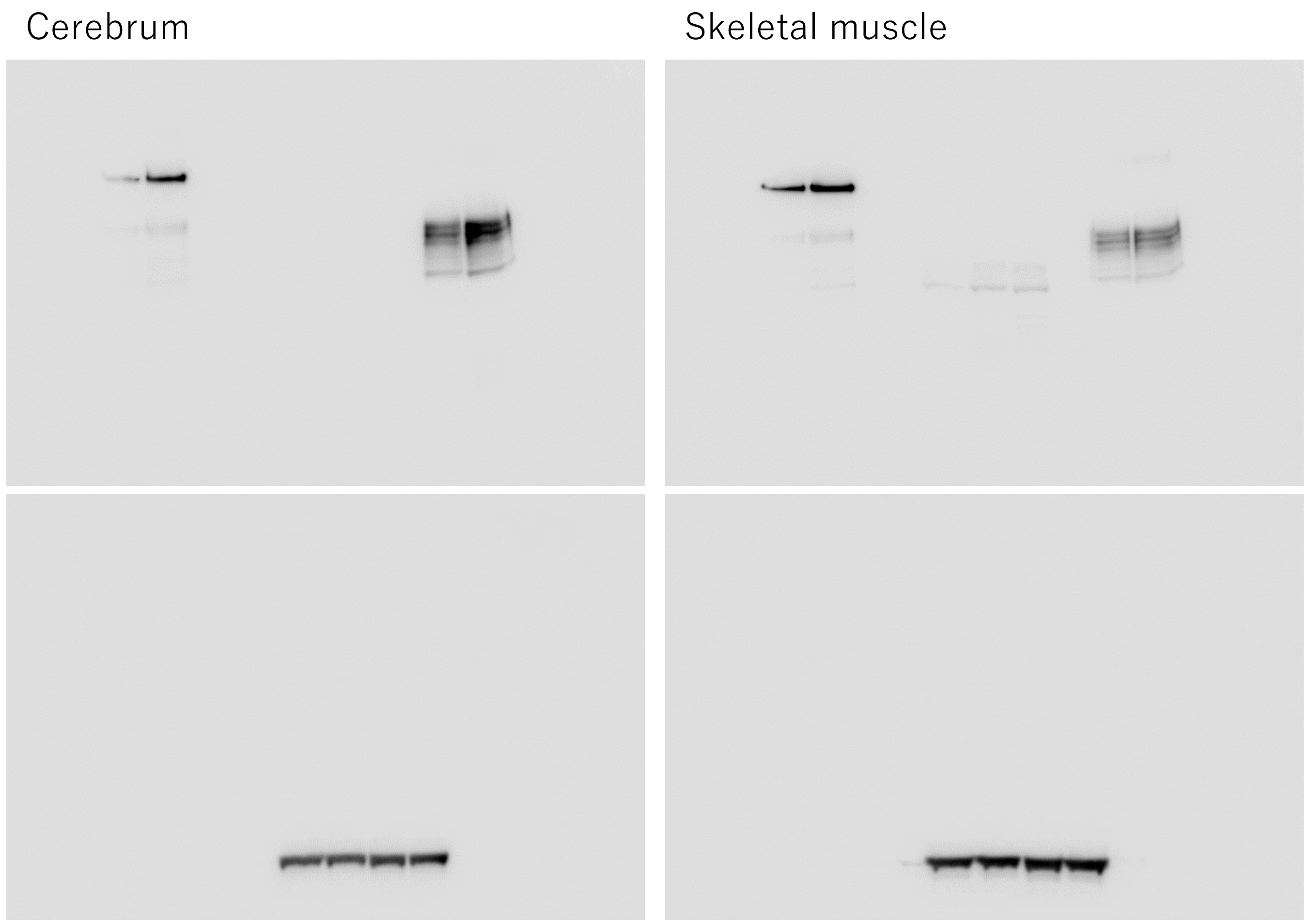

Supplement Figure2B

Lung

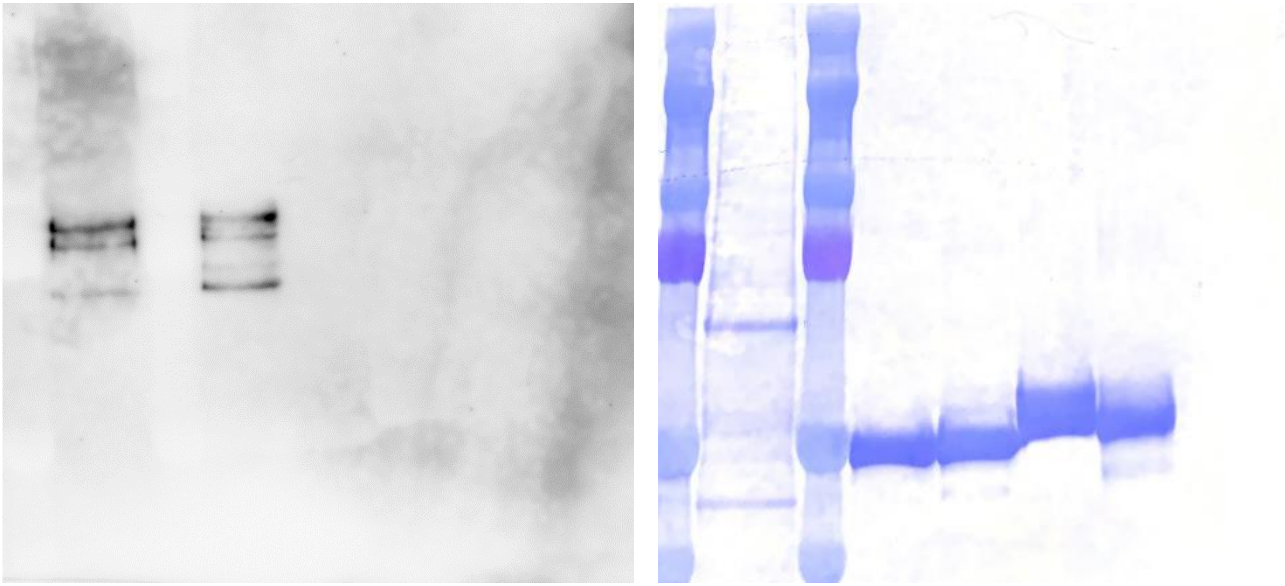

Colon

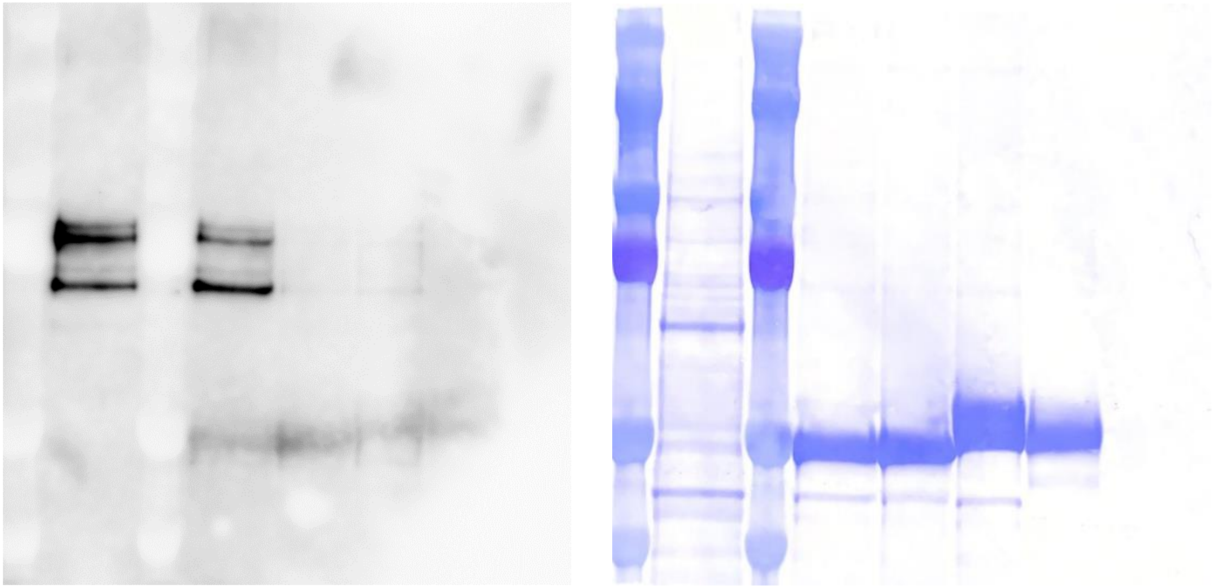

Supplement: Supplementary file 1 [file biomolecules-14-01093-s001.zip › western blotting original images.pdf]
